# The role of the dentist and orthodontist in recognizing oro-facial manifestations of acromegaly: a questionnaire-based study

**DOI:** 10.1007/s11102-021-01183-y

**Published:** 2021-09-13

**Authors:** Giorgia Preo, Alberto De Stefani, Francesca Dassie, Alexandra Wennberg, Roberto Vettor, Pietro Maffei, Antonio Gracco, Giovanni Bruno

**Affiliations:** 1 Faculty of Dentistry, Padua University, Padua, Italy; 2 DIMED, Padua University, Padua, Italy; 3 Unit of Epidemiology, Institute of Environmental Medicine, Karolinska Institutet, Stockholm, Sweden

**Keywords:** IGF-1, GH, Oral-disorders, Dental manifestations, Prognathism, Macroglossia

## Abstract

**Purpose:**

Oro-facial manifestations of acromegaly are among the earliest signs of the disease and are reported by a significant number of patients at diagnosis. Despite this high prevalence of acromegaly oral manifestation, dentists do not play a pivotal role in acromegaly identification and diagnosis. The aim of our study was to evaluate the ability of dentists and orthodontists in the early recognition of the oro-facial manifestations of acromegaly.

**Methods:**

A telematic questionnaire was administered to dentists and orthodontists. The questionnaire included photos with facial and oral-dental details and lateral teleradiography of acromegaly patients (ACRO).

**Results:**

The study included 426 participants: 220 dentists and 206 orthodontists. Upon reviewing the photos, dentists most often observed mandibular prognathism and lips projection, while orthodontists also reported the impairment of relative soft tissue. Orthodontists, who usually use photos to document patients’ oral-facial characteristics, paid more attention to oral-facial impairment than dentists. During dental assessment, 90% of the participants usually evaluated tongue size and appearance, diastemas presence, and signs of sleep impairment (mainly orthodontists). Orthodontists were also more able to identify sella turcica enlargement at teleradiography. A total of 10.8% of the participants had ACRO as patients and 11.3% referred at least one patient for acromegaly suspicion.

**Conclusion:**

The study highlighted dentists’ strategic role in identifying ACRO. Increasing dentists’ awareness about acromegaly clinical issues may improve early diagnosis, potentially resulting in an increased quality of life and decreased mortality among ACRO.

**Supplementary Information:**

The online version contains supplementary material available at 10.1007/s11102-021-01183-y.

## Introduction

Acromegaly is a multisystemic disease characterized by somatic changes, such as acral overgrowth, brow prominence, prognatism, macroglossia, enlargement of lips and nose, and a wide range of comorbidities, such as cardiovascular and metabolic manifestations [1–4]. Due to the slow onset of symptoms, patients affected by acromegaly experience a delay in diagnosis that may range from 5 to more than 10 years, with detrimental effects on comorbidity progression and quality of life [2, 3, 5–7].

Facial dysmorphism and oro-dental alterations are among the most common and earliest manifestations of acromegaly [8–10]. The appearance of facial dysmorphisms can precede acromegaly diagnosis by up to 10 years [11]. Facial features of acromegaly patients (ACRO) include mandibular prognathism, development of supraorbital ridges and cheekbones, alteration in the size of the nose and ears, thickening of soft tissues and lips, and asymmetry of the face [2, 5, 12, 13]. Additionally, up to 80% of these patients present with oral characteristics, including mandibular growth (22–24%), bite impairment, such as a dental class III [2, 5, 8, 12, 14], dental malocclusion, dental diastema (40–43%) [2, 5, 8], and macroglossia (54–58%) [2, 8, 14], all of which may contribute to the development of obstructive sleep disorders [2, 8, 14].

In the literature it is well known that despite the high prevalence of facial dysmorphism and oro-dental alterations, dentists and orthodontists do not play a pivotal role in acromegaly suspicion and diagnosis. In their everyday clinical practice, these specialists have the habit of documenting patients’ clinical characteristics with photos and dental X-rays; moreover, they diagnose and treat oral-facial impairments. Therefore, dentists and orthodontists are well-suited to detect early changes of oral-facial characteristics and to follow these patients’ features over time. We believe that dentists and orthodontists can play a critical front-line role in identifying ACRO. To confirm this hypothesis and to increase awareness of ACRO features and comorbidities among dentists, we created a specific questionnaire on acromegaly oral-facial characteristics. The primary goal of our study was to evaluate the role of dentists and orthodontists in recognizing the oro-facial manifestations of acromegaly and therefore evaluate their potential in the early diagnosis of the disease. The secondary end point was to highlight whether there were any differences in the results between general dentists and orthodontists.

## Methods

The study was carried out at the Dental Clinic at the University Hospital of Padua in collaboration with the UOC Clinica Medica III in the Medicine Department at the University Hospital of Padua.

We conducted a literature search on oral-facial manifestations of acromegaly using Pubmed and Google Scholar databases. We included the following keywords: “*acromegaly”, “acromegaly AND dentistry”, “oral-disorders AND acromegaly”, “facial manifestations of acromegaly”, “dental manifestations AND acromegaly”, “oral diagnostician AND acromegaly”, “dentist AND acromegaly”, “acromegaly oro-dental diagnosis”, “dentofacial structure AND acromegaly”, “clinical features of acromegaly”, “growth hormone”, “prognathism”, “macroglossia”, and “sella turcica”*. Based on the most frequent oral-facial manifestations of acromegaly described in literature, a questionnaire on oral-facial impairment of ACRO was formulated. The questionnaire was controlled by two Endocrinologists expert on acromegaly disease.

The questionnaire was sent in electronic format to Italian dentists and orthodontists in the Dental Clinic mailing list and was signed by the research group in orthodontics of the University of Padua. The questionnaire was proposed as research on the theme of *facial harmony*, because the pathognomonic aspects of the syndrome had to be identified without suggestions. The final questionnaire included ten sections. The first section aims to investigate the type of clinical practice. The second section aimed to understand the importance of oral-facial characteristics analysis and the type of investigations used to study oral-facial manifestations in everyday clinical practice among dentists and orthodontists. In sections three, four, and five, identify in the images which facial features of these patients were less harmonious and which could be the expression of an altered systemic condition. Sections six, seven, and eight focus on the clinical practice evaluation of the most common acromegaly oral impairment and comorbidities, such as macroglossia and tongue evaluation, diastema in the lower arch, and sleep disorders. Section nine compared radiographic examinations of acromegalic patients and control patients. The final section (ten) presents specific questions on acromegaly and reveals the real aim of the questionnaire. At the end of the questionnaire, a summary and explanatory text was presented, with the aim of collecting the prevailing aspects of the research and of the topic, mainly to raise awareness among dentists and orthodontists about acromegaly. In addition, it was necessary for the respondents to answer all the questions in order to complete and send the answers of the questionnaire.

Questionnaire photos of ACRO and controls were collected at Orthodontic Therapy out-service at the Dental Clinic of the Hospital of Padua. Controls were patients followed for a dental and skeletal class III impairment. We collected photos of 19 ACRO. According to acromegaly clinical guidelines, diagnosis of acromegaly was based on previous laboratory and pituitary MRI findings, data were collected from patients’ charts [15]. Each patient was clinically examined and photographic documentation was collected: photos of the face in frontal and in profile projection with a relaxed expression and closed mouth, frontal and lateral intraoral photos, photos for the evaluation of the oral lingual encumbrance and photos of oral anatomical details (such as diastemas or malocclusions). A black background was used to standardize the documentation and the anatomical details of the patients' faces were obscured to ensure anonymity. The aim of the examination was to evaluate and document the characteristics of acromegaly in these patients. The tele-radiographic examination of the acromegalic patient included in the questionnaire was obtained from the literature [16].

## Statistic analysis

Numerical data were summarized as median and interquartile range (IQR), while categorical data as absolute frequency (n) and relative frequency (%). The comparison between dentists and orthodontists was carried out with the Mann–Whitney test (numerical data), the Chi Square test (categorical data) and the Fisher test (categorical data). A p-value of less than 0.05 was considered statistically significant. All analyses were completed with R 4.0 software (R Foundation for Statistical Computing, Vienna, Austria).

## Results

The study included 426 Italian clinicians (220 dentists and 206 orthodontists). Answers to the questionnaire are shown in Table 1. Data regarding participants and their clinical practice showed that dentists were younger (p = 0.010) with fewer years of experience (p = 0.003) than orthodontists. The prevalent work environment was similar between groups, and most of the respondents said they work in the private sector (Table 1, Section 1). Regarding clinical practice, orthodontists paid greater attention than dentists in the collection of patients’ faces photos (p < 0.0001), in the analysis of the anatomo-aesthetic characteristics (p < 0.0001), in the use of facial analysis software (p < 0.0001), in the observation of the health conditions of the perioral soft tissues and of the face (p < 0.0001), and in observation of the general anatomical characteristics of patients (p < 0.0003) (Table 1, Section 2).

**Table 1:** Main results of the questionnaire

Variables	Participants number 426	General dentists 220 (51.6)	Orthodontists 206 (48.4)		p-value
Section 1					
Age					**0.01**
25–35 years	151 (35.4)	89 (40.4)	62 (30.1)		
36–45 years	100 (23.5)	38 (17.3)	62 (30.1)		
46–55 years	84 (19.7)	47 (21.4)	37 (18.0)		
Over 55 years	91 (21.4)	46 (21.0)	45 (21.8)		
Working experience					**0.003**
< 5 years	92 (21.6)	62 (28.2)	30 (14.6)		
5–10 years	69 (16.2)	32 (14.5)	37 (18.0)		
> 10 years	265 (62.2)	126 (57.3)	139 (67.5)		
Main work area					0.510
Hospital	16 (3.8)	9 (4.1)	7 (3.4)		
Private	392 (92.0)	204 (92.7)	188 (91.3)		
University	18 (4.2)	7 (3.2)	11 (5.3)		
Section 2					
Collects and keeps photos of patients' faces	351 (82.4)	147 (66.8)	204 (99.0)		** < 0.001**
Analyzes the anatomical-aesthetic characteristics of the patients' face for diagnostic and therapeutic purposes	376 (88.3)	174 (79.1)	202 (98.0)		** < 0.001**
Uses software dealing with facial analysis	153 (35.9)	43 (19.5)	110 (53.4)		** < 0.001**
Observes the state of health of the perioral soft tissues and of the face	362 (85.0)	171 (77.7)	191 (92.7)		** < 0.001**
Looks at the general anatomical characteristics of the patients, as well as those of the face and oral cavity					** < 0.001**
No	44 (10.3)	33 (15.0)	11 (5.4)		
Yes	314 (73.7)	145 (65.9)	169 (82.0)		
Only in growing patients	68 (16.0)	42 (19.1)	26 (12.6)		
Section 5					
Less harmonic characteristics (see supplementary material section 5)					
Prognathism	363 (85.1)	185 (84.1)	178 (86.4)		0.590
Lip anatomy	224 (52.6)	111 (50.5)	113 (54.9)		0.420
Projection of the cheekbones	93 (21.8)	40 (18.2)	53 (25.7)		0.080
Appearance of soft tissues	82 (19.2)	29 (13.2)	53 (25.7)		**0.002**
Projection of supraorbital ridges	61 (14.1)	30 (13.6)	31 (15.0)		0.780
Nasal projection	52 (12.2)	32 (14.5)	20 (9.7)		0.170
Facial biotype	37 (8.7)	24 (10.9)	13 (6.3)		0.130
Section 6					
Considers the appearance and size of the patients' tongues	374 (87.8)	191 (86.8)	183 (88.8)		0.630
It is common, in their clinical practice, to find language with altered dimensions (in excess)					0.850
No	141 (33.1)	75 (34.1)	66 (32.0)		
Yes	261 (61.3)	132 (60.0)	129 (62.6)		
I do not know	24 (5.6)	13 (5.9)	11 (5.3)		
In their clinical practice, it is common to ask patients questions about the quality of their sleep, snoring, daytime sleepiness, or nocturnal breathing problems					** < 0.001**
No	87 (20.4)	60 (27.3)	27 (13.1)		
Yes	274 (64.3)	110 (50.0)	164 (79.6)		
Only to adults	33 (7.7)	29 (13.2)	4 (1.9)		
Only to pediatric patients	32 (7.6)	21 (9.5)	11 (5.4)		
In clinical practice, they have referred a patient to investigate sleep (for any complaints such as sleep apnea)					**0.002**
No	138 (32.4)	89 (40.4)	49 (23.8)		
Yes	252 (59.1)	111 (50.5)	141 (68.4)		
Only to adults	23 (5.4)	13 (5.9)	10 (4.9)		
Only to pediatric patients	13 (3.1)	7 (3.2)	6 (2.9)		
Section 9					
Identify in the image what, in your opinion, is a sign of a pathology (more than one response is acceptable)					
III skeletal class	370 (86.9)	194 (88.2)	176 (85.4)		0.490
The conformation of the sinus	53 (12.4)	22 (10.0)	31 (15.0)		0.150
The conformation of sella turcica	211 (49.5)	86 (39.1)	125 (60.7)		** < 0.001**
The appearance of the condyles	125 (29.3)	54 (24.5)	71 (34.5)		**0.030**
The conformation of the cervical vertebrae	76 (17.8)	49 (22.3)	27 (13.1)		**0.020**
Section 10					
Their patients include individuals with acromegaly					**0.009**
No	343 (80.5)	169 (76.8)	174 (84.5)		
Yes	46 (10.8)	23 (10.5)	23 (4.4)		
I do not remember	37 (8.7)	28 (12.7)	9 (11.1)		

Data expressed as n (%)

Significant p-values are in Bold

The anatomical details of the face of an ACRO that were most often perceived by the interviewees as a sign of an altered systemic condition were mandibular prognathism and the appearance of the lips. The appearance of the soft tissues of the face was more often evaluated by orthodontists than by dentists (Table 1, Section 3). Moreover, on Section 3 of the questionnaire, dentists and orthodontists reported that they usually did not recognize specific differences between patients and controls. Indeed, none of the dentists identified any of the acromegaly patients as affected by a systemic condition. Only two acromegaly patients were identified as affected by systemic condition: one was recognized by 84.7% of the participants (general dentists vs orthodontists: 83.1% vs 86.4%, p = NS) whereas 35.7% of interviewees identified the other (general dentist vs orthodontists: 35.9% vs 35.4%, p = NS); there were no statistically significant differences between dentists and orthodontists in the responses.

Ninety percent of the participants stated that they commonly consider the appearance and size of the tongue of patients in clinical practice and that they encounter altered-sized tongues (61.3%), with no differences between dentists and orthodontists. In the clinical practice of the participants, the presence of diastemas in the lower arch was frequently reported (69%). For the purposes of a diagnostic-therapeutic evaluation, 96.5% of the participants (general dentists vs orthodontists: 95.9% vs 97.1%, p = NS) considered diastemas a clinical significant sign and they considered important an anamnestic investigation regarding its onset. This happened to 74.4% of the participants. Most (87.3%) of the participants considered the increase in size of the jaw as clinically significant and 35.4% reported a communication with patients on this issue. Communication was more frequent among orthodontists than dentists (41.3% vs 30%, p < 0.05). Over half (56.8%) of the participants also said they propose a solution to the increase in size of the jaw in an adult patient when it is observed. 31.9% of the interviewees would refer the patients to a non-dental specialist without significative differences between dentists and orthodontists (34.6% of general dentists vs 29.1% of orthodontists, p = NS).

Approximately half (54.7%) of the participants reported receiving frequent facial pain complaints and 92.2% considered patient-reported headache as a symptom in their dental evaluations, with no statistically significant difference between general dentists and orthodontists (respectively 94.1% vs 90.3%, p = NS).

Two-thirds (64.3%) of the participants stated that they inquired about the sleep quality of patients, with orthodontists reporting doing so more frequently than dentists (79.6% vs 50.0% respectively, p < 0.05) and 59.1% of the participants referred some patients to do more in-depth sleep examinations, orthodontists more frequently than dentists (68.4% vs 50.0% respectively, p < 0.05) (Table 1, Section 6).

In the evaluation of an acromegalic patient’s radiological teleradiography in latero-lateral projection, the orthodontists more often identified the anatomical alteration at the level of the sella turcica (p < 0.05) (Table 1, Section 9).

About one-tenth of the respondents reported having patients with acromegaly, dentists more frequently than orthodontists (Table 1, Section 10), and 11.3% of the participants reported having sent at least one patient for acromegaly testing.

## Discussion

The literature does not identify dentists as specialists who have a particular role in the diagnostic pathway of ACRO, despite the potential of their clinical position. Our study aimed to evaluate dentists’ ability to recognize the oro-facial signs of acromegaly. The study revealed that orthodontists could actually be the clinicians most involved in detecting ACRO earlier, as their training leads them to focus on evaluating the anatomical-facial characteristics of patients. The identification of the radiological anatomical alterations of the sella turcica also highlighted an important ability of orthodontists.

Acromegaly diagnosis is often delayed due to the insidious nature of this disease. In a recent study, the *Liège Acromegaly Survey*, involving more than 3000 patients, authors found that acromegaly was most frequently identified by endocrinologists (44.9%), general practitioners/family doctors (17.5%), or internists (13.2%). Rheumatologists/orthopedic specialists (3.6%), neurologists (3.3%) and ophthalmologists (2.3%) are involved to a lesser extent. It also emerged that in 2.3% of cases the diagnosis was made by the patient themselves or by their family/friends [17]. The variety of non-endocrinological specialists who diagnose acromegaly is significant, however, dentists do not seem to have an important role in this regard, which is echoed in other studies [18–21]. In our study, dentists and orthodontists did not recognize all the acromegaly patients reported in the photos. About one-tenth of participants referred at least one patient for acromegaly suspicion and 10% of this cohort reported that they visit patients with acromegaly in their clinics. Our results therefore seem to be in line with those reported in the literature, in which dentists do not seem to have a fundamental role in the diagnostic path of these patients, despite a long term follow-up with a collection of photographic and radiographic documentation. In the literature, it is well known [18, 20] that dentists are among the clinicians most consulted by patients before diagnosis and these data emphasize the potential role of this medical practitioner, who could be the first specialist to notice the early signs of acromegaly [22, 23]. Moreover, another study [8] points out that despite a significant percentage of acromegalic patients evaluated for dental care prior to diagnosis, less than 5% of these are referred to a specialist due to suspected acromegaly. The strategic role of the dentist is confirmed by the following studies [24, 25], which note the importance of accuracy in the evaluation and updating of the patient's ongoing medical history.

The results of this study also showed that the most evident aspects of the disease for dentists seem to be the mandibular prognathism and the projection of the lips, anatomical characteristics closely related to the oral cavity that are also pathognomonic signs of acromegaly. Some studies in literature [8, 26] revealed that about 80% of the ACRO under examination had oral-dental-facial disorders, in particular macroglossia (54–58%), dental diastema (40–43%), mandibular growth (22–24%), and mandibular prognathism (20–22%). Another study confirmed that [18] oral cavity and face somatic changes in acromegaly patients are present up to 10 years before diagnosis. In particular, it emphasized how facial changes are among the most common signs, being the third most frequent alteration at diagnosis, immediately after the increase in the size of feet and hands. The presence of diastemas also plays an important diagnostic role for another study, which included this sign in the entries of the system for the diagnosis of acromegaly ACROSCORE, thus considering it a pathognomonic sign of the disease [11].

Moreover, our study shows that dental X rays could also be used to suspect acromegaly. Eighty-seven percent and 50% of interviewees identify the III skeletal class and impaired conformation of sella turcica, respectively, as a sign of systemic pathology. Our data are in line with those of literature, in fact, in our cohort, orthodontists are more prone to identify changes in appearance of soft tissue and collateral findings on dental X-ray.

Early diagnosis of acromegaly significantly improves patients' quality of life and reduces the risk of mortality. The role of early diagnosis is significant: it has been shown that if the disease is identified within 2 years of its onset, the clinical pictures show milder characteristics [26], including oral-facial disorders and, in particular for macroglossia, diastemas and prognathism [8]. However, the literature reveals that it is still rare for the diagnosis of acromegaly to be made within one year of the onset of the first symptoms, despite medical advances [18]. In this context, dentists and, in particular, orthodontists who provide continuous and long-term care to patients and have access to radiographic/photographic follow-ups and to facial analysis software such as those used in orthodontic programs or by scanner devices [25], are critical for early diagnosis. According to some articles, orthodontists should play a more important role, as they usually document and check patients’ photos [26, 27]. The collected photos of patients can also be used for early diagnosis of acromegaly using artificial intelligence methods. In particular, Kong et al. highlighted how machine learning methods can recognize with high sensitivity and specificity the facial features of acromegaly, helping physicians during acromegaly screening and follow-up [28]. Artificial intelligence plays an increasingly important role in medicine and could be applied to screen clinical settings and a recent review summarized different artificial intelligence solutions adopted in literature that showed the ability to early detect acromegaly with good sensitivity and specificity [29].

Dentists and orthodontists may also have a role in the diagnosis of acromegaly comorbidities. In particular, one of the most frequent comorbidities of acromegaly is the sleep apnea syndrome (OSA). In acromegaly, OSA prevalence ranges between 20 and 80% [30] and it can be present in active and inactive patients. OSA may be underestimated at diagnosis and during follow-up. Two-thirds of the clinicians in this study usually ask patients about sleep disorders, snoring, and daytime sleepiness and often refer patients for a sleep quality assessment. These data are very important because a correct evaluation of sleep disorder risk in ACRO and the early identification of OSA could improve patient quality of life and reduce their mortality [31].

A limit of this study is that despite the real focus of the questionnaire was revealed at the end it was possible to go back to previous questions. However, we don’t think that this aspect could have affected the diversity of answers between dentists and orthodontists. In conclusion, dentists could play a strategic role in identifying patients with acromegaly and their comorbidities, however, the fact that only a very small percentage of them have claimed to have intercepted the suspects suggests that there is not yet enough knowledge and sensitivity with respect to the pathology. Raising the awareness of dentists on clinical issues of patients with acromegaly, the availability of tool as ACROSCORE for non-endocrinological specialists, and the use of photos with patients’ facial and oral details may improve acromegaly early diagnosis.

## Supplementary Information

Below is the link to the electronic supplementary material.

Supplementary file1 (DOCX 6780 kb)

## Data Availability

Data are available upon reasonable request.
