# Supplementary material for: The role of the dentist and orthodontist in recognizing oro-facial manifestations of acromegaly: a questionnaire-based study
Source: Pituitary. 2021 Sep 13;25(1):159–66. doi: 10.1007/s11102-021-01183-y (PMC8821049; doi:10.1007/s11102-021-01183-y)
Supplement: Supplementary file 1 — Supplementary file1 (DOCX 6780 kb) [file 11102_2021_1183_MOESM1_ESM.docx]

**QUESTIONNAIRE ABOUT FACIAL ARMONY**

**SECTION 1**

- What do you mainly deal with in your clinical practice? (possibility to select more than one answer)

Oral surgery, implantology and oral pathology

Conservative dentistry and endodontics

Dental prosthesis

Orthodontics and gnathology

Pedodontics

- Please indicate your age

Between 25 and 35 years old

Between 36 and 45 years old

Between 46 and 55 years old

Over 55 years old

- How many years have you been practicing as a dentist?

For less than 5 years

Between 6 and 10 years

For over 10 years

- You work mainly at a

Hospital

Private clinic

University

**SECTION 2**

- In your clinical practice, do you usually collect and store photos of your patients' faces?

Yes

No

- In your clinical practice, is it usual to analyze the anatomical and aesthetic characteristics of the face of your patients for diagnostic and therapeutic purposes?

Yes

No

- Is it usual, in your clinical practice, to use a facial analysis software?

Yes

No

- In your clinical practice, do you usually analyze the health conditions of the perioral and facial soft tissues (such as lips, nose, cheekbones)?

Yes

No

- In your clinical practice, do you usually observe the general anatomical characteristics of your patients?

Yes

No

Yes, but only in growing patients

**SECTION 3**

- In your opinion, which of these faces are the least “harmonious” (possibility to select more than one answer)?


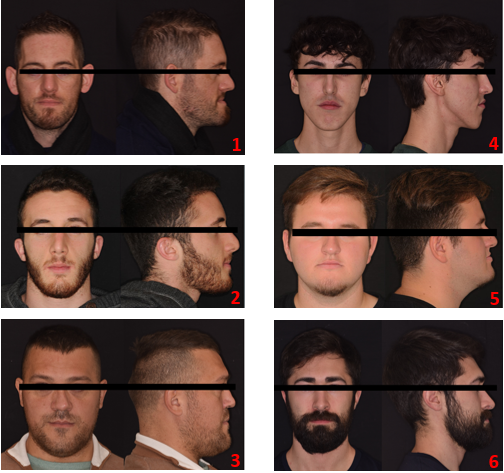


Option 1

Option 2

Option 3

Option 4

Option 5

Option 6

- In your opinion, which of these subjects could be affected by an altered systemic condition (possibility to select more than one answer)?

Option 1

Option 2

Option 3

Option 4

Option 5

Option 6

**SECTION 4**

- Please indicate, on a scale from 1 to 10, how "harmonious" this face seems to you


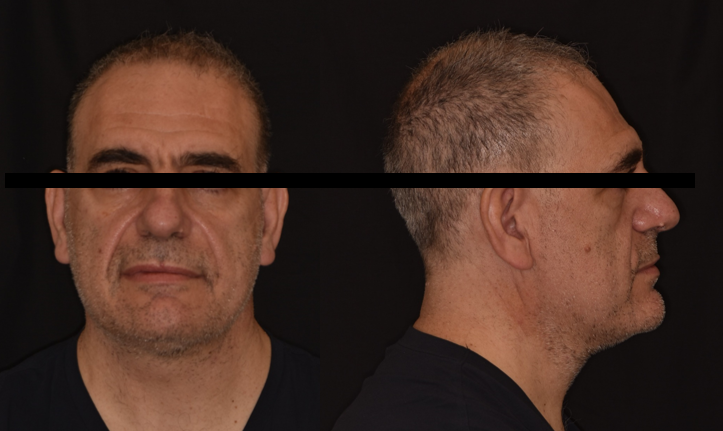


Not harmonious o o o o o o o o o o harmonious

- Please indicate, on a scale of 1 to 10, how "harmonious" this face seems to you


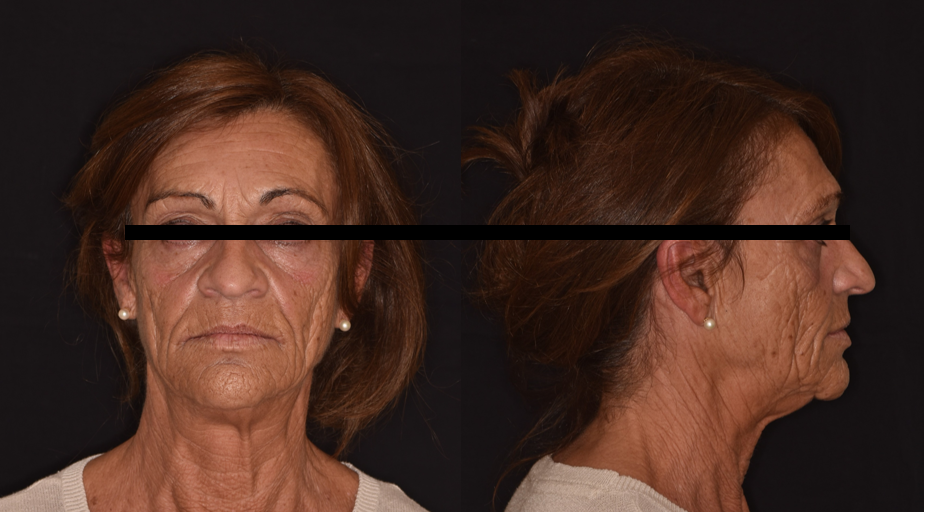


Not harmonious o o o o o o o o o o harmonious

- Please indicate, on a scale of 1 to 10, how "harmonious" this face seems to you


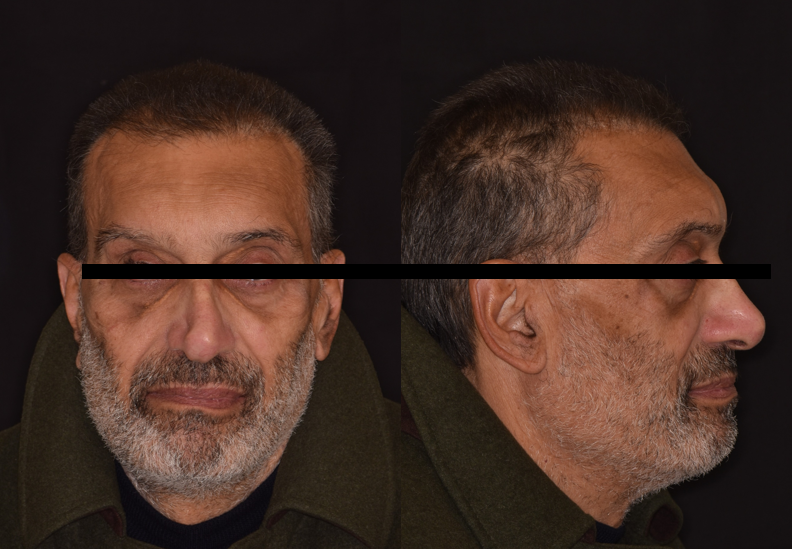


Not harmonious o o o o o o o o o o harmonious

- Please indicate, on a scale of 1 to 10, how "harmonious" this face seems to you


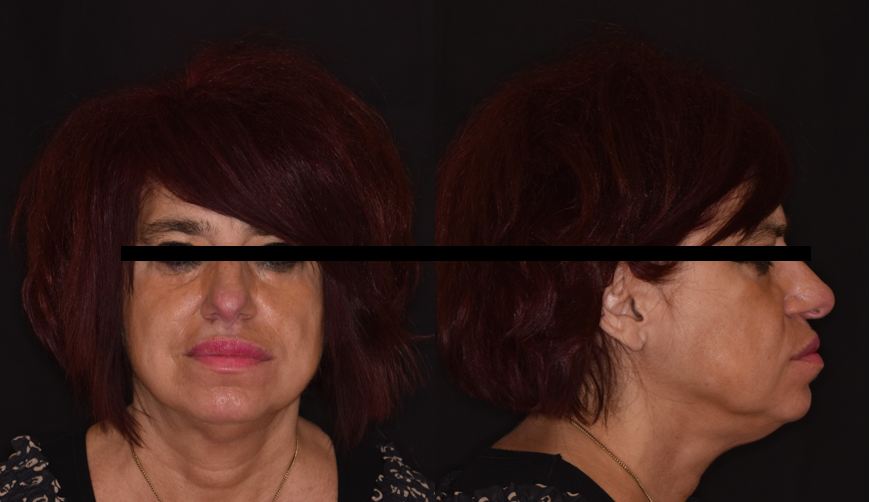


Not harmonious o o o o o o o o o o harmonious

- Please indicate, on a scale of 1 to 10, how "harmonious" this face seems to you


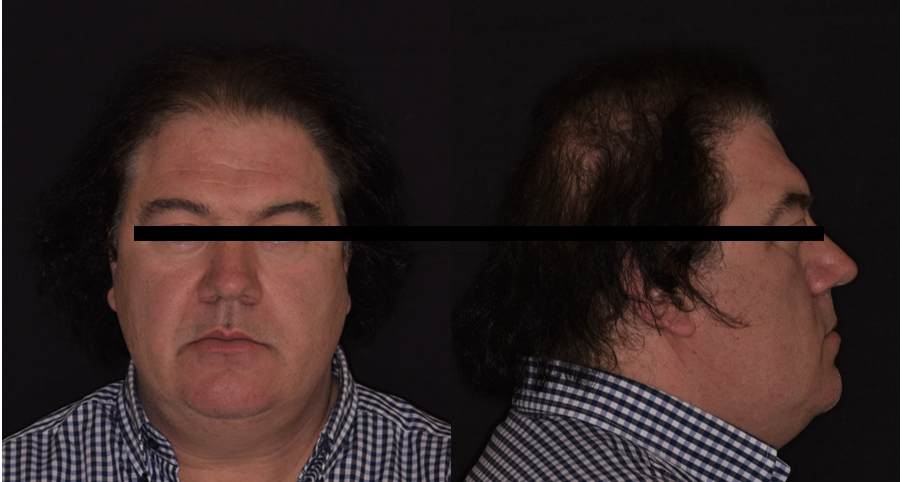


Not harmonious o o o o o o o o o o harmonious

- Please indicate, on a scale of 1 to 10, how "harmonious" this face seems to you


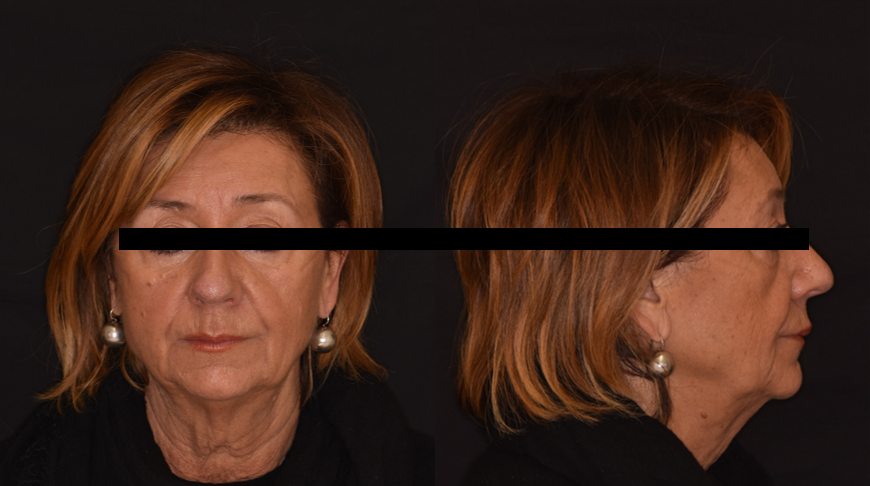


Not harmonious o o o o o o o o o o harmonious

- In your opinion, which of these subjects could be affected by an altered systemic condition (possibility to select more than one answer)?


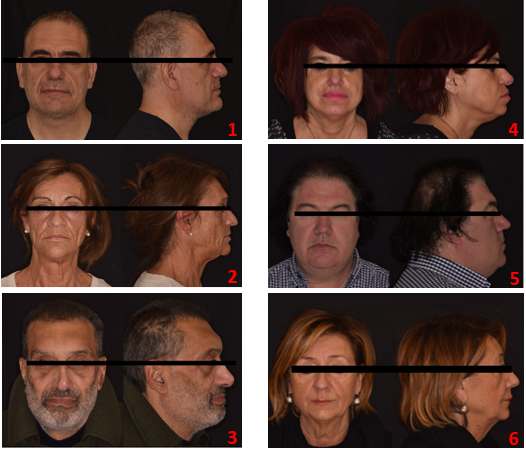


Option 1

Option 2

Option 3

Option 4

Option 5

Option 6

**SECTION 5**

- Which anatomical characteristics of this photo appear to you "not harmonious" or catch your attention (possibility to select more than one answer)?


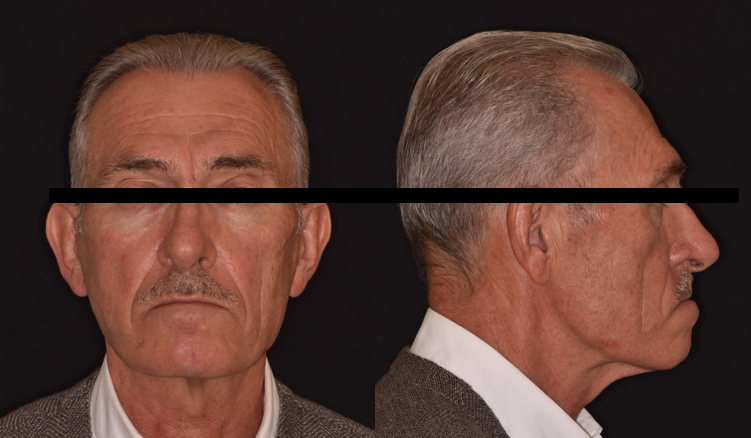


The prognathism

The nasal projection

The supraorbital ridges projection

The anatomy of the lips

The facial biotype

The appearance of soft tissues

The cheekbones projection

**SECTION 6**

- Is it usual, in your clinical practice, to consider the appearance and the size of the patient's tongue?

Yes

No

- Is it common, in your clinical practice, to find patients with an altered tongue (excessive size)?


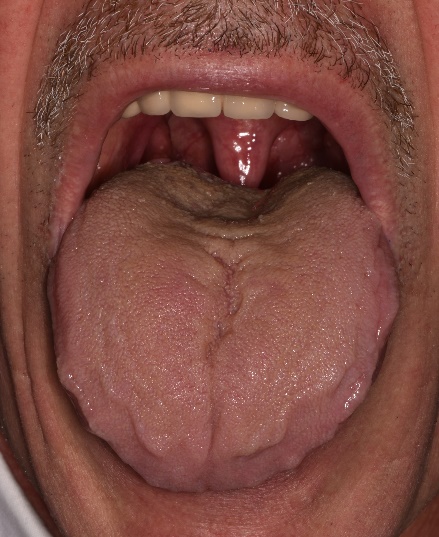


Yes

No

I don’t know

- Is it usual, in your clinical practice, to ask patients questions about the quality of their sleep, snoring, daytime sleepiness, or nocturnal breathing problems?

Yes

No

Yes, but only for adults

Yes, but only for pediatric patients

- In your clinical practice, have you ever referred a patient to a specialist, to undergo some tests for sleep disorders (for example for sleep apneas)?

Yes

No

Yes, but only for adults

Yes, but only for pediatric patients

- In your opinion, if a patient tells you that he or she has started snoring for no apparent reason, would this information be an aspect to be investigated?

Yes

No

Depends on the age of the patient

**SECTION 7**

- In your clinical practice, is it common to find diastemas in the lower arch?


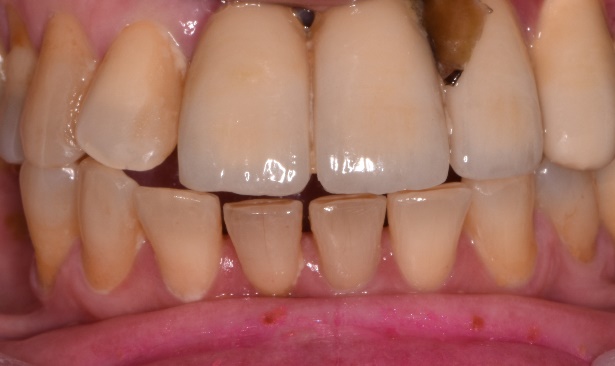


Yes

No

I don’t know

- In the event that an adult patient reported to you that up to 10 years earlier their teeth did not present such diastemas, would you consider the information useful and significant for the purposes of a diagnostic-therapeutic evaluation?

Yes

No

- Have you ever had a patient that reported such information (referring to the previous question)?

Yes

No

- In the event that an adult patient told you that his jaw seems to have increased in size, would you consider this statement significant for the purpose of a diagnostic-therapeutic evaluation?

Yes

No

- Have you ever had a patient reporting such information (referring to the previous question)?

Yes

No

- In your clinical practice, would you propose a solution to this condition (referring to the previous question)?

No

Yes, orthodontic / surgical solution

Yes, by referring the patient to another non-dental specialist

**SECTION 8**

- In your clinical practice, is it common to receive patients who complain of facial pain?

Yes

No

- Do you usually consider the headache as a symptom in your dental evaluations?

Yes

No

**SECTION 9**

- In your opinion, do these two radiographs represent the same problem?


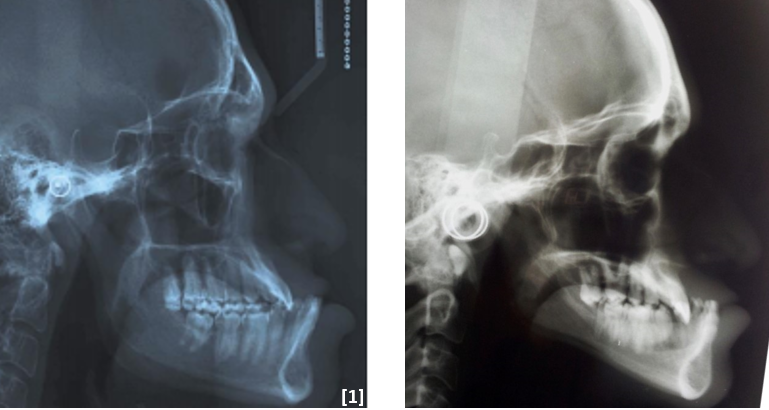


Yes

No

- What do you consider, in your opinion, a sign of pathology in this image (you can choose more than one answer)?


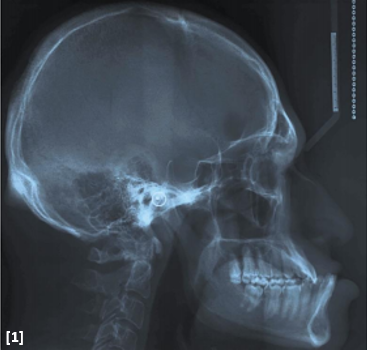


The III skeletal class

The conformation of the sinus

The conformation of the sella turcica

The condyles

The conformation of the cervical vertebrae

**SECTION 10**

- Are there any patients with acromegaly among your patients?

Yes

No

I do not remember

- In your opinion, what are the epidemiological data of this pathology?

10-20 cases / million inhabitants

90-100 cases / million inhabitants

About 500 cases / million inhabitants

About 1000 cases / million inhabitants

- In your opinion, is this pathology easy to identify for a dentist?

Yes

No

I don’t know

- In your personal experience, have you ever referred a patient to a specialist to inspect this pathology?

Yes

No

**REFERENCES**

[1] T. Freundlich, D. Arueste, G. Manríquez, and A. Díaz, “Comparación cefalométrica entre un paciente acromegálico y su hermano gemelo Cephalometric comparison between an acromegalic patient and his twin brother,” vol. 33, 2019.
